# Controlled PolyDMAEMA Functionalization of Titanium Surfaces via Graft-To and Graft-From Strategies

**DOI:** 10.3390/mi16080899

**Published:** 2025-07-31

**Authors:** Chiara Frezza, Susanna Romano, Daniele Rocco, Giancarlo Masci, Giovanni Sotgiu, Monica Orsini, Serena De Santis

**Affiliations:** 1 Department of Industrial, Electronic and Mechanical Engineering, Roma Tre University, Via Vito Volterra 62, 00146 Rome, Italy; chiara.frezza@uniroma3.it (C.F.); susanna.romano@uniroma3.it (S.R.); daniele.rocco@uniroma3.it (D.R.); giovanni.sotgiu@uniroma3.it (G.S.); monica.orsini@uniroma3.it (M.O.); 2 Department of Chemistry, Sapienza University, Piazzale A. Moro 5, 00185 Rome, Italy; giancarlo.masci@uniroma1.it

**Keywords:** titanium functionalization, PDMAEMA brushes, graft-to/graft-from strategies, stimuli-responsive polymers

## Abstract

Titanium is widely recognized as an interesting material for electrodes due to its excellent corrosion resistance, mechanical strength, and biocompatibility. However, further functionalization is often necessary to impart advanced interfacial properties, such as selective ion transport or stimuli responsiveness. In this context, the integration of smart polymers, such as poly(2-(dimethylamino)ethyl methacrylate) (PDMAEMA)—noted for its dual pH- and thermo-responsive behavior—has emerged as a promising approach to tailor surface properties for next-generation devices. This work compares two covalent immobilization strategies for PDMAEMA on titanium: the “graft-to” method, involving the attachment of pre-synthesized polymer chains, and the “graft-from” method, based on surface-initiated polymerization. The resulting materials were characterized with size exclusion chromatography (SEC) for molecular weight, Fourier-transform infrared spectroscopy (FTIR) for chemical structure, scanning electron microscopy (SEM) for surface morphology, and contact angle measurements for wettability. Electrochemical impedance spectroscopy and polarization studies were used to assess electrochemical performance. Both strategies yielded uniform and stable coatings, with the mode of grafting influencing both surface morphology and functional stability. These findings provide valuable insights into the development of adaptive, stimuli-responsive titanium-based interfaces in advanced electrochemical systems.

## 1. Introduction

Titanium (Ti) is recognized as a material of choice for electrodes in a wide range of electrochemical applications, owing to its unique combination of physicochemical properties. Its high corrosion resistance, mechanical strength, and biocompatibility make it particularly suitable for both biomedical devices [1] and advanced energy systems [2,3]. The exceptional passivation behavior of titanium, attributed to the spontaneous formation of a stable TiO_2_ layer, provides robust protection against aggressive environments, such as those encountered in electrolysis, fuel cells, and biomedical implants [4,5,6]. This intrinsic stability not only prolongs device lifespan but also ensures consistent electrochemical performance, even under prolonged operational stress. Beyond its inherent stability, titanium offers good electrical conductivity and a relatively low density, making it advantageous for applications where both structural integrity and lightweight design are essential. In the context of renewable energy and next-generation electrochemical devices, titanium electrodes are increasingly investigated for their ability to support high current densities without significant degradation, as well as their compatibility with a wide range of surface modification strategies [7,8,9].

Functionalization of titanium surfaces is often required to impart additional functionalities, such as selective ion transport, antifouling behavior, or stimuli-responsiveness. In this regard, the integration of smart polymers onto titanium surfaces has emerged as a promising approach to tailor interfacial properties for specific applications. Among the various candidates, poly(2-(dimethylamino)ethyl methacrylate) (PDMAEMA) stands out due to its dual pH- and thermo-responsive behavior, as well as its tunable physicochemical characteristics. PDMAEMA is a cationic, water-soluble polymer that exhibits reversible changes in conformation and solubility in response to environmental stimuli [10]. At low pH, the tertiary amine groups along the PDMAEMA backbone become protonated, rendering the polymer highly hydrophilic and positively charged. Conversely, at higher pH values, deprotonation leads to a more hydrophobic and collapsed conformation. This pH-responsiveness is complemented by a lower critical solution temperature (LCST) behavior, whereby PDMAEMA undergoes a coil-to-globule transition upon heating above 30–50 °C, depending on pH, molecular weight, concentration, and ionic strength, further modulating its surface properties [11,12]. These features make PDMAEMA highly attractive for applications such as controlled drug delivery, antifouling coatings, and, more recently, functional layers in electrochemical devices. In addition to these well-established uses, PDMAEMA brushes grafted onto titanium surfaces offer the intriguing possibility of acting as molecular machines, dynamic, stimulus-responsive systems capable of reversible and controllable changes at the molecular level [13]. The reversible coil-to-globule transitions and pH-induced charge modulation can be harnessed to actively regulate surface interactions, ion transport, and molecular accessibility in real time. These conformational changes enable the brush layer to function as a nanoscale actuator, capable of switching between extended and collapsed states in response to external stimuli. Such behavior mimics key features of artificial molecular machines, enabling the design of responsive interfaces for advanced applications, including smart implants, switchable membranes, and adaptive electrochemical systems.

In this context, the method of PDMAEMA immobilization plays a critical role in determining the stability and performance of the resulting composite material. Traditional deposition techniques, such as physical adsorption or layer-by-layer assembly, often exhibit poor long-term stability, particularly under harsh electrochemical conditions. Desorption, delamination, or gradual degradation of the polymer layer can compromise device performance and limit practical applicability. In this work, we focused on the covalent attachment of PDMAEMA chains to titanium surfaces using either a “graft-to” or “graft-from” strategy. In the graft-to approach, pre-synthesized PDMAEMA chains bearing reactive end groups are chemically linked to functionalized titanium surfaces. In contrast, the graft-from method utilizes surface-immobilized initiators to enable the direct polymerization of DMAEMA monomers from the substrate. This dual strategy enabled a direct comparison of the two most widely adopted approaches for polymer grafting, providing valuable insights into their relative effectiveness in producing uniform, stable, and well-defined polymer coatings. The synthesized PDMAEMA was characterized with gel permeation chromatography (GPC), confirming that both graft-to and graft-from methods yielded polymers with comparable molecular weights and low dispersity. Surface morphology and coverage were investigated using scanning electron microscopy (SEM), while wettability was assessed via contact angle measurements. Electrochemical performance was evaluated through impedance spectroscopy and polarization studies, demonstrating the functional integrity and stability of the coatings.

## 2. Materials and Methods

### 2.1. Materials

Titanium foils (thickness 0.127 mm, 99.7% trace metals basis), sodium azide (≥99.5%), (+)-sodium l-ascorbate (crystalline, ≥98%), potassium carbonate (K_2_CO_3_), copper (II) sulfate pentahydrate (98%), copper(I) chloride (CuCl), copper(II) chloride (CuCl_2_), 2,2′-Bipyridine (bpy), 2-(Dimethylamino)ethyl methacrylate (DMAEMA) and propargyl bromide were purchased from Merk Life Science, Milano, Italy. Hydrochloric acid (HCl 37%), sodium hydroxide (NaOH, 98%), and DMF were supplied from Carlo Erba Reagents, Milano, Italy. All reagents were used as received.

### 2.2. Titanium Surface Pretreatment

Commercially pure titanium foil was sectioned into pieces measuring approximately 1.5 × 2 cm^2^. Each sample underwent cleaning by sequential ultrasonic baths: first in a 1:1 (*v*/*v*) mixture of water and acetone, followed by ethanol, each for 10 min. Subsequently, the samples were rinsed thoroughly with distilled water and dried using gentle airflow at room temperature. For acid etching, the titanium pieces were immersed in an 18 wt% hydrochloric acid solution maintained at 80 °C for 10 min. Base etching was carried out by soaking the samples in a 5 M sodium hydroxide solution at 80 °C for 2 h. After these treatments, the samples were rinsed repeatedly with distilled water until the pH of the rinsing solution reached neutrality. To ensure the removal of any residual debris, the samples were further cleaned in an ultrasonic bath: first with distilled water for 5 min, then with ethanol for an additional 5 min. Finally, the samples were dried under a stream of air at ambient temperature.

### 2.3. Surface Functionalization via Graft-To Method

**Figure 1:**
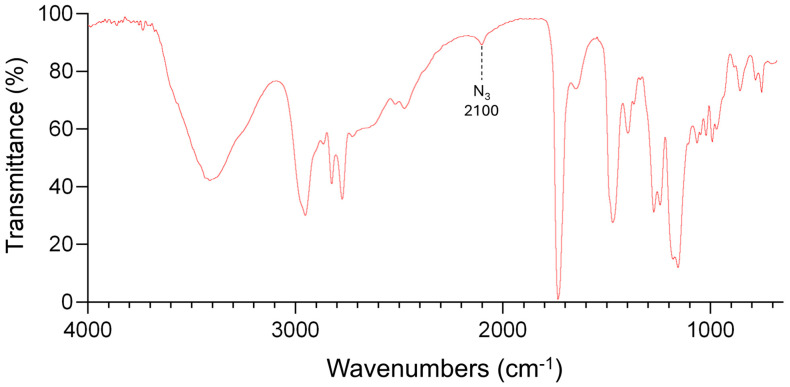
FTIR spectrum of N_3_-PDMAEMA.

N_3_-PDMAEMA was synthesized following a previously reported procedure [14]. Briefly, Atom Transfer Radical Polymerization (ATRP) was performed using N-(2-azidoethyl)-2-bromo-2-methylpropanamide (αBIB-N_3_) as the initiator. The polymerization was carried out in a 1:1 (*v*/*v*) mixture of DMF and water at 40 °C, with a feed ratio of [DMAEMA]/[αBIB-N_3_]/[CuCl]/[CuCl_2_]/[2,2′-bipyridine] = 50/1/4/0.8/9.6 and a DMAEMA concentration of 3 M. CuCl, CuCl_2_, and 2,2′-bipyridine were placed in a Schlenk tube and subjected to three cycles of evacuation and argon backfilling to remove oxygen. Half of the solvent and monomer, both previously degassed with argon, were then added, and the mixture was stirred to equilibrate. In a separate vial, the αBIB-N_3_ initiator was dissolved in the remaining solvent and monomer, which were also degassed. This solution was then introduced into the Schlenk tube to initiate polymerization. A sample was immediately withdrawn to determine the initial monomer concentration (t = 0), and monomer conversion during the reaction was monitored using HPLC. Additional aliquots were collected at selected times for molecular weight analysis with SEC. The polymerization was terminated by bubbling air through the reaction mixture for 10 min. The resulting solid polymer was purified via extensive dialysis (initially against acidified distilled water, then against distilled water) to obtain the polymer in its protonated form. Finally, the product was recovered by lyophilization. Figure 1 shows the FTIR spectrum of the synthesized PDMAEMA-N_3_ displaying the characteristic absorption bands of PDMAEMA, including C=O stretching around 1730 cm^−1^ and C–H stretching near 2850–2950 cm^−1^. In addition, a distinct peak at approximately 2100 cm^−1^ confirms the presence of the azide functional group.

Hydroxyl groups on NaOH pre-treated titanium samples were subjected to a nucleophilic substitution reaction with propargyl bromide in H_2_O/acetone at 100 °C for 24 h, in the presence of K_2_CO_3_ as the base to give the Ti functionalized propargyl derivative [15].

Finally, the titanium surface was functionalized via the copper(I)-catalyzed azide-alkyne Huisgen 1,3-dipolar cycloaddition (CuAAC, or “click” reaction) following the procedure described in Marsotto et al. [16]. Briefly, PDMAEMA-N_3_ was dissolved in distilled water and stirred overnight. Sodium ascorbate and copper(II) sulfate were then added to the solution under an argon atmosphere to generate the active copper(I) catalyst. The alkyne-functionalized titanium samples were immersed in this reaction mixture and maintained at 40 °C with stirring for 72 h. After the reaction, the samples were thoroughly rinsed with distilled water and cleaned by sequential ultrasonic baths in a water/acetone mixture and ethanol to remove any unreacted species or residues.

### 2.4. Surface Functionalization via Graft-From Method

The radical initiator α-bromoisobutyryl bromide (BIBB) was anchored onto the pretreated titanium surfaces following a previously reported procedure [17]. Briefly, the titanium specimens were placed in a 150 mL round-bottom flask equipped with a condenser and a magnetic stir bar. A solution containing BIBB, bipyridine, and dichloromethane (CH_2_Cl_2_) was added, and the mixture was refluxed at 70 °C for 30 min. After the reaction, the specimens were removed and thoroughly rinsed with CH_2_Cl_2_ and EtOH to remove unreacted reagents and impurities. The surface-initiated ATRP was carried out by adapting the previously described protocol, with the addition of ethyl 2-bromo-2-methylpropionate (EBIB) in solution as a sacrificial initiator. This allowed for controlled polymer growth from the surface, enabling molecular weight control and characterization of the free polymer chains formed in solution.

### 2.5. Size Exclusion Chromatography (SEC)

Molecular weight distributions were analyzed using a size exclusion chromatography (SEC) system equipped with a LabFlow 4000 HPLC pump (LabService Analytica, Bologna, Italy) and two Polymer Laboratories PLgel Mixed-B columns (Agilent Technologies, Santa Clara, CA, USA,10 μm particle size, 300 × 7.5 mm), maintained at 60 °C using a Waters Millipore TCM column oven (Milford, MA, USA). Detection was performed with a Shimadzu RID-10A refractive index detector (Shimadzu, Kyoto, Japan). The mobile phase consisted of DMF containing 5% *v*/*v* triethylamine (TEA), flowing at 0.8 mL/min. Number–average molecular weight (Mn) and dispersity (Mw/Mn) values were calculated by calibration against near-monodisperse polymethylmethacrylate standards. Polymer samples were prepared at a concentration of 1 mg/mL, and an injection volume of 100 μL was used for each analysis.

### 2.6. Fourier-Transform Infrared Spectroscopy (FTIR)

Fourier transform infrared (FT-IR) spectra were acquired using a Nicolet iN10 FTIR spectrometer (Thermo Scientific, Waltham, MA, USA) equipped with a liquid nitrogen-cooled MCT detector, a spectral range from 4000 to 675 cm^−1^. For each sample, at least 10 measurements were acquired from different areas. Each FTIR-ATR spectrum was an average of 64 scans, with 8 cm^−1^ resolution. Spectral data were processed using the OMNIC 9.13.1256 Nicolet iN10 microscopy software package (Thermo Scientific, Waltham, MA, USA).

### 2.7. Scanning Electron Microscopy (SEM) and Energy-Dispersive X-Ray Microanalysis (EDS)

The morphology of the titanium surfaces was examined using scanning electron microscopy (SEM) on a Zeiss Gemini Sigma 300 field-emission gun (FEG) SEM (Jena, Germany). Images were captured at an accelerating voltage of 5 kV, utilizing a backscattered electron detector, with a working distance set to 7.5 mm. The chemical composition of the samples was examined with energy-dispersive X-ray microanalysis (EDS, INCAx-sight, model: 7426, Oxford Instruments, Abingdon, UK) at a reduced accelerating voltage of 5 kV, with a backscattered detector and a working distance of 8 mm.

### 2.8. Atomic Force Microscopy (AFM)

Atomic Force Microscopy (AFM) analyses were conducted using a Dimension Icon system (Bruker AXS, Billerica, MA, USA) under ambient air and room temperature conditions in tapping mode. Scans covered an area of 40 × 40 µm^2^. High-resolution RTESP (Rotated Tapping Etched Silicon) probes (VEECO Probes, Camarillo, CA, USA) featuring sharp tips with a manufacturer-specified radius of curvature of approximately 8 nm were employed. These probes were mounted on rectangular cantilevers with a length of 125 µm, a resonant frequency of 300 kHz, and a spring constant of 40 N/m. Image processing was performed using the open-source software Gwyddion version 2.28 (http://gwyddion.net/), applying only background subtraction and flattening to correct for image tilt and offsets.

### 2.9. Contact Angle Measurements

Contact angle measurements were conducted using the sessile drop technique with an Attension Theta Optical Tensiometer (Biolin Scientific, Gothenburg, Sweden). Each measurement lasted 3 s, during which 125 to 140 images were captured and analyzed. The instrument automatically defined the baseline throughout the measurement. The contact angle (θ) was calculated by fitting the droplet profile to the Young–Laplace equation.

### 2.10. Electrochemical Measurements

Electrochemical characterization was conducted using Na_2_SO_4_ as the electrolyte, as it provides a stable, low-interference environment for surface-sensitive measurements. The corrosion behavior of the coated samples was evaluated with potentiodynamic polarization using an AMEL System 5000 workstation (AMEL, Milan, Italy). Data acquisition was performed using CorrWare software version 3.5c (Scribner Associates Inc., Southern Pines, NC, USA), and data analysis was conducted with CorrView software version 3.5c (Scribner Associates Inc., Southern Pines, NC, USA). Tests were conducted on 1 cm^2^ sample areas at room temperature in a 0.1 M Na_2_SO_4_ aqueous electrolyte. A three-electrode electrochemical cell was employed, with the titanium samples serving as the working electrode (WE), a platinum wire as the counter electrode (CE), and an Ag/AgCl electrode as the reference. Polarization scans were performed at a rate of 10 mV/s relative to the open-circuit potential (OCP), over a potential range of −400 mV to +500 mV. Electrochemical impedance spectroscopy (EIS) measurements were performed under similar experimental conditions using a Metrohm Autolab PGSTAT204 (Metrohm Autolab B.V., Utrecht, The Netherlands) instrument. The tests spanned a frequency range from 100 kHz to 10 mHz, applying an AC perturbation of ±10 mV around the open-circuit potential (OCP).

## 3. Results and Discussion

### 3.1. Surface Activation and Functionalization

**Figure 2:**
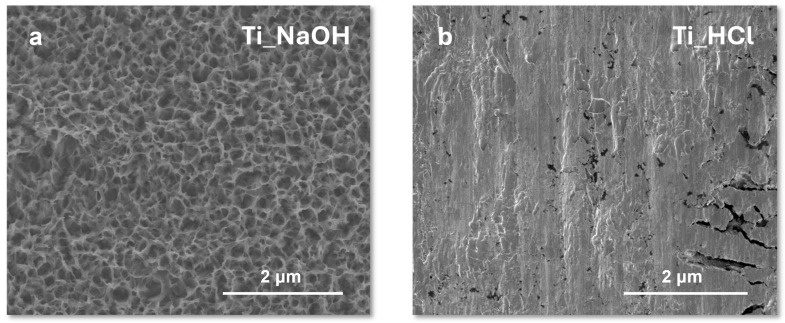
SEM images of the titanium NaOH (**a**) and HCl (**b**) treated surfaces.

The initial step in the functionalization of titanium surfaces involved activating the metal substrate through a controlled etching process. As already demonstrated in a previous study [18], pre-treatment can significantly improve subsequent grafting reactions, as it directly influences the availability and nature of reactive groups on the titanium surface. To optimize the surface activation, two different etching protocols were compared: acidic etching with hydrochloric acid (HCl) and basic etching with concentrated sodium hydroxide (NaOH). SEM images of the treated surfaces are shown in Figure 2.

As discussed in previous work from our laboratory [16], SEM images of titanium surfaces etched with concentrated NaOH revealed the formation of a highly porous architecture characterized by interconnected nanopores. This nanostructuring is attributed to the aggressive alkaline attack, which promotes the dissolution and reprecipitation of titanium oxide, resulting in a sponge-like morphology with a high density of accessible surface area. In contrast, surfaces treated with HCl displayed irregularly distributed deep pits. The acidic environment leads to localized dissolution of the oxide layer, creating uneven, crater-like features across the surface. Both NaOH and HCl pre-treatments were systematically tested to identify which treatment would best prepare the titanium for each specific grafting strategy, namely, the graft-to approach via click chemistry and the graft-from approach via SI-ATRP. The effectiveness of the grafting was evaluated using FTIR spectroscopy, specifically by analyzing the presence and intensity of the characteristic PDMAEMA signals on the treated surfaces (cfr par. 3.3). For the graft-to approach, which requires the initial functionalization of the titanium surface with 1-bromo-2-propyne, the pre-treatment with concentrated NaOH proved to be more effective. The alkaline etching promotes the formation of a highly hydroxylated and roughened titanium oxide layer, thereby enhancing the nucleophilicity of the surface and facilitating subsequent reactions with alkyne-functionalized bromides. This results in a higher density of reactive sites, ultimately improving the efficiency of the click chemistry-based grafting of pre-synthesized PDMAEMA chains. Conversely, acidic pre-treatment with HCl was found to be superior for the graft-from approach, where the titanium surface is functionalized with α-bromo isobutyryl bromide to introduce ATRP initiator groups. Acidic etching results in a smoother, cleaner oxide surface with a high density of accessible –OH groups, which are ideal for the covalent attachment of bromoisobutyryl moieties. These assessments of better suitability are based on FTIR spectroscopy results, where the alternative pre-treatment for each grafting method showed weak or absent characteristic PDMAEMA signals, indicating lower grafting efficiency.

**Scheme 1:**
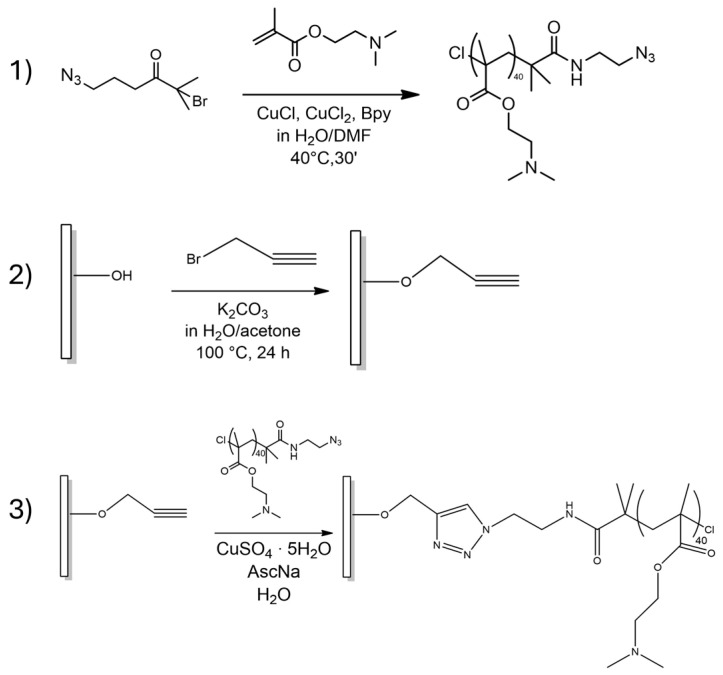
Titanium surface polymerization with PDMAEMA40 using grafting-to approach. 1) azide-terminated PDMAEMA is synthesized via ATRP. 2) the titanium surface, previously pretreated with NaOH, is functionalized with propargyl bromide to introduce alkyne terminal groups. 3) the PDMAEMA chains are grafted onto the alkyne-functionalized titanium surface through CuAAC.

Following surface activation, the functionalization reactions were carried out using two distinct strategies. In the graft-to protocol (Scheme 1), pre-formed PDMAEMA chains bearing azide end-group (N_3_-PDMAEMA) were covalently attached to the alkyne-functionalized titanium via copper-catalyzed azide-alkyne cycloaddition (“click” chemistry). The alkyne end-functionalized titanium surface was obtained by attaching propargyl bromide onto the NaOH-treated surface, while ATRP synthesis of PDMAEMA was performed in DMF–water mixtures with a method previously used for solid phase synthesis [14]. 

**Scheme 2:**
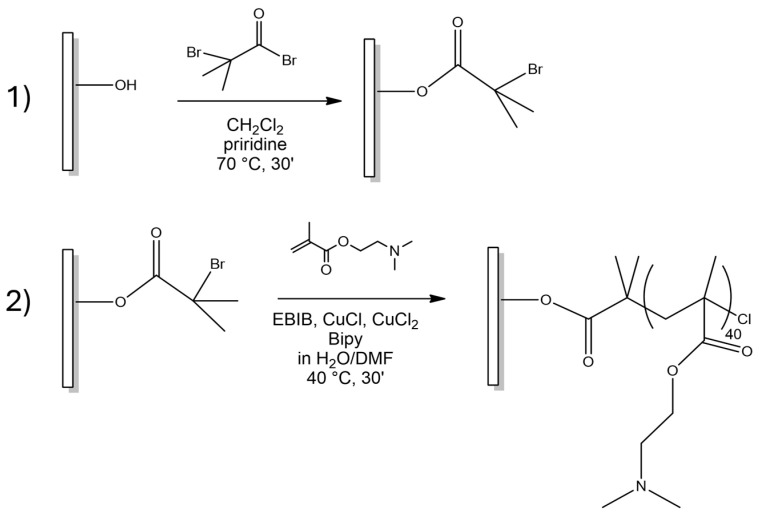
Titanium surface polymerization with PDMAEMA_40_ using grafting-to approach. 1) The titanium surface, previously pretreated with HCl, is functionalized with α-bromoisobutyryl bromide to introduce surface-bound ATRP initiator groups. 2) surface-initiated-ATRP) PDMAEMA is carried out in the presence of EBIB as a sacrificial initiator.

In the graft-from protocol (Scheme 2), the titanium surface, after initiator immobilization, was used as a substrate for direct polymerization of DMAEMA via Surface Initiated ATRP (SI-ATRP).

After the α-bromoisobutyryl bromide ATRP initiator was inserted into the acidic pretreated surface, the ATRP polymerization was conducted under the same conditions described for the alternative approach. Polymerization was conducted in the presence of ethyl 2-bromo-2-methylpropionate (EBIB), structurally resembling the surface-bound initiator, as the sacrificial initiator. The addition of a sacrificial initiator in SI-ATRP compensates for the low concentration of surface-bound initiators, ensuring that the molecular weight and dispersity of grafted polymers closely match those of polymers synthesized simultaneously in solution under identical conditions [19].

Samples obtained using the graft-to-graft and graft-from approaches are identified in the text as Ti_click and Ti_ATRP, respectively.

### 3.2. Polymer Characterization with Size Exclusion Chromatography (SEC)

**Figure 3:**
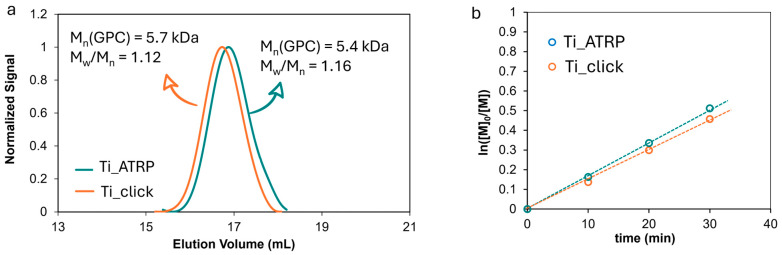
(**a**) GPC trace of the obtained polymer; (**b**) First-order kinetic plot (top) for ATRP of DMAEMA in DMF/H_2_O 50:50 (*v*/*v*), [DMAEMA]/[αBIB-N_3_]/[CuCl]/[CuCl_2_]/[BPy] = 100/1/4/0.8/9.6 (orange) and [DMAEMA]/[αBIB]/[CuCl]/[CuCl_2_]/[BPy] = 100/1/4/0.8/9.6 (blue), at 40 °C.

A fundamental objective of this study was to synthesize PDMAEMA chains with comparable molecular weights and dispersities for both the graft-to and graft-from approaches. This equivalence enables a direct comparison of the resulting surface properties, thereby minimizing the influence of polymer chain length and distribution on the observed differences. For the graft-to method, the N_3_-PDMAEMA was characterized with size exclusion chromatography (SEC), which revealed a number-average molecular weight (Mn) of 5.7 kDa and a narrow polydispersity index (PDI) of 1.12 (Figure 3a).

These values confirm a well-controlled polymerization, yielding relatively low molecular weight chains suitable for surface immobilization. SEC analysis of the polymer formed in solution during SI-ATRP showed an Mn of 5.4 kDa and a dispersity of 1.16, closely matching the graft-to polymer (Figure 3a). Both polymerizations exhibited linear kinetic profiles, indicative of their living or controlled character (Figure 3b). This behavior ensures that the polymer chain ends remain active, thus enabling further functionalization steps such as chain extension or copolymerization. The relatively low molecular weight chosen for these experiments facilitates the investigation of subtle differences introduced by the two functionalization strategies at early stages, before the effects of chain entanglement or steric hindrance become significant.

### 3.3. Surface Characterization with FTIR

**Figure 4:**
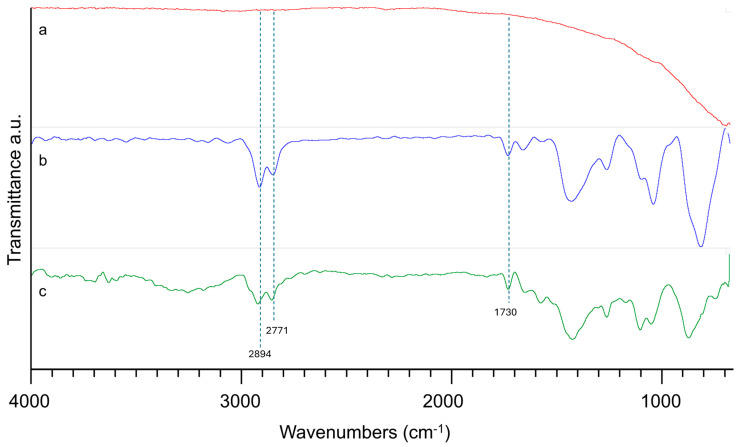
FTIR spectra of (**a**) unmodified titanium substrate, (**b**) titanium surface functionalized via click chemistry, and (**c**) titanium surface functionalized via surface-initiated atom transfer radical polymerization (SI-ATRP).

The functionalized titanium surfaces were analyzed with Fourier-transform infrared spectroscopy (FTIR) following a rigorous cleaning protocol consisting of 20 min of sonication and thorough washing to remove any physically adsorbed species. For both graft-to and graft-from samples, characteristic absorption bands attributable to PDMAEMA were consistently detected across multiple points on each surface, confirming the successful covalent attachment of the polymer (Figure 4).

Specifically, the FTIR spectrum of unmodified titanium (Figure 4a) shows no significant absorption bands in the analyzed range, confirming the absence of organic functional groups on the surface. In contrast, both Ti_click and Ti_ATRP (Figure 4a and b, respectively) display clear signals corresponding to PDMAEMA, including the ester C=O stretching at 1730 cm^−1^, C–H stretching around 2894 cm^−1^ and 2771 cm^−1^, as well as characteristic bands in the fingerprint region between 1268 and 853 cm^−1^. This spatially uniform detection of polymer signals across the sample surface highlights the homogeneity of the functionalization.

As a control experiment, non-functionalized titanium samples were immersed in the respective reaction solutions under identical conditions, followed by the same sonication and washing procedures. FTIR analysis of these control samples showed no detectable polymer-related signals, demonstrating that the detected PDMAEMA bands on the functionalized samples arise from covalently bound polymer layers rather than from residual physically adsorbed material. This confirms the robustness of the grafting protocols and the stability of the polymer coatings against rigorous cleaning, supporting their suitability for further application.

### 3.4. Surface Morphology: SEM and AFM Analysis

**Figure 5:**
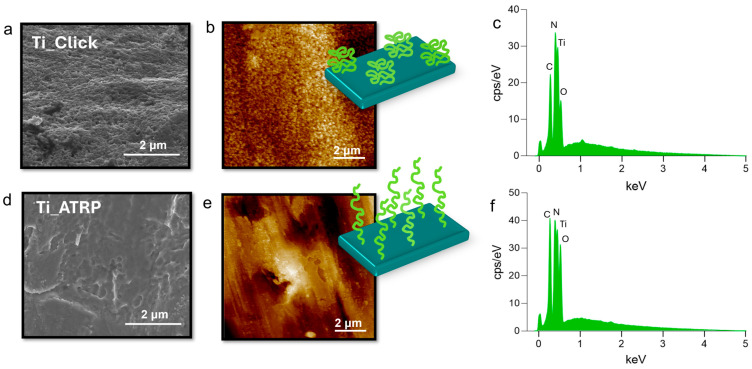
SEM (**a**,**d**), AFM (**b**,**e**), and EDS (**c**,**f**) images of the titanium surfaces after functionalization. Top row (**a**–**c**) corresponds to the Ti_click samples, while bottom row (**d**–**f**) shows the Ti_ATRP samples.

The morphological evolution of titanium surfaces following different pre-treatment and functionalization strategies was systematically investigated using scanning electron microscopy (SEM) and atomic force microscopy (AFM). SEM and AFM micrographs are shown in Figure 5.

SEM and AFM analyses of the PDMAEMA-functionalized surfaces, prepared via click chemistry and SI-ATRP techniques, revealed distinct topographies that reflected both the initial surface structure and the intrinsic differences in grafting density between the two methods. Surfaces prepared using the graft-to approach exhibited a pronounced granularity, with the appearance of contiguous, rounded features resembling closely packed “beads”. This morphology arises because of a relatively low grafting density, allowing individual polymer chains sufficient space to adopt a globular conformation, similar to their behavior in dilute solution [20,21]. In contrast, the surfaces functionalized via SI-ATRP presented a much more compact and uniform appearance. The higher grafting density achieved with this technique forces the polymer chains to grow predominantly in the direction perpendicular to the substrate, resulting in a dense, brush-like layer [22]. This vertical orientation and close packing of the chains produce a smoother, more homogeneous surface at the nanoscale, as confirmed with both SEM and AFM analyses.

EDS analysis was also performed at a reduced acceleration voltage of 5 keV to limit the beam’s penetration and thus accentuate the sensitivity to signals from the polymer coating, while minimizing the signal from the underlying titanium substrate. The signals for carbon, nitrogen, and oxygen, together with those from titanium, are entirely consistent with the expected elemental composition of the PDMAEMA coating. In particular, the presence of a prominent nitrogen peak, clearly distinguishable despite partial overlap with the adjacent titanium signal, confirms the presence of the PDMAEMA layer.

### 3.5. Surface Wettability: Contact Angle Measurements

**Figure 6:**
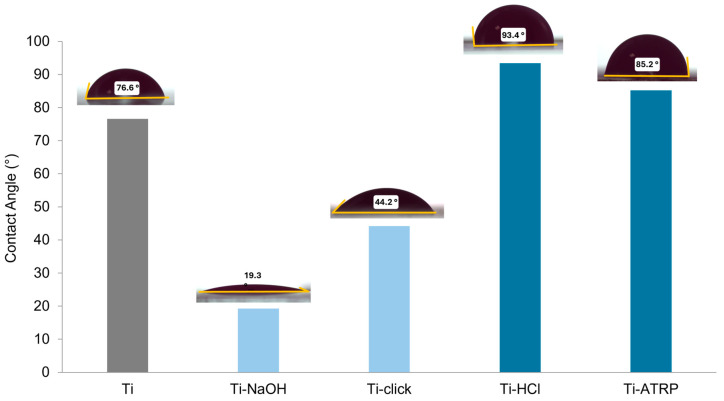
Contact angle measurements for unmodified titanium (Ti), NaOH-treated titanium (Ti–NaOH), alkyne-functionalized titanium after click reaction (Ti_click), HCl-etched titanium (Ti–HCl), and ATRP-functionalized titanium (Ti_ATRP).

Contact angle (CA) measurements provide direct insight into the wettability of titanium substrates before and after functionalization. The data illustrate the progressive changes in surface wettability resulting from chemical and polymeric surface modifications (Figure 6).

The pre-treatment step significantly alters the wettability of titanium. HCl etching increases the contact angle to 116.9°, indicating a significant increase in hydrophobicity. In contrast, NaOH etching decreases the contact angle to 43.7°, indicating a highly hydrophilic surface due to the introduction of numerous –OH groups. The click-functionalized surface exhibits a contact angle of ~44.2°, closely matching that of the hydrophilic NaOH-treated substrate. The coiled conformations of the PDMAEMA chains result in greater exposure of their hydrophilic tertiary amine groups—which are partially protonated under the neutral pH conditions of the experiment [23]—to the aqueous environment, thereby lowering the contact angle. In Ti_ATRP, the denser packing can reduce the accessibility of hydrophilic groups at the interface, thereby attenuating their hydrophilicity. Nevertheless, the SI-ATRP surface shows a decreased CA value (~85.2°) compared to the pretreatment, indicating partial restoration of hydrophilicity.

Importantly, the relatively short chain lengths used in this work (Mn~5.5 kDa) form thin layers where the underlying substrate’s surface chemistry and morphology features, established by the pre-treatment, still significantly affect wettability. As chain length and brush thickness increase, the substrate influence diminishes, and contact angles are expected to converge toward values characteristic of bulk PDMAEMA films, which may exhibit different wettability due to polymer chain packing and hydration. However, while thicker polymer brushes are often preferred for applications such as surface protection [24] and self-cleaning surfaces [25], utilizing shorter polymer chains can be advantageous for applications that require precise control over surface morphology and facilitate further chemical modifications. Short brushes, in fact, maintain higher chain-end accessibility and mobility, which can be exploited for post-functionalization or stimuli-responsive behavior [26]. Moreover, thin polymer layers preserve nanoscale substrate features, which may be critical in applications such as biosensing or catalysis. In such contexts, the surface-bound polymer layer can be designed to expose or conceal functional motifs in response to external stimuli, thereby enabling on-demand modulation of cell adhesion, catalytic activity, or enzymatic recognition—a characteristic particularly relevant for the development of biointerfaces and adaptive surface coatings.

The contact angle data reflect a complex interplay between polymer chain conformation, grafting density, chain length, and substrate effects. Understanding and controlling these parameters is essential for tailoring surface wettability and optimizing the performance of polymer-functionalized titanium interfaces.

### 3.6. Electrochemical Characterization

**Figure 7:**
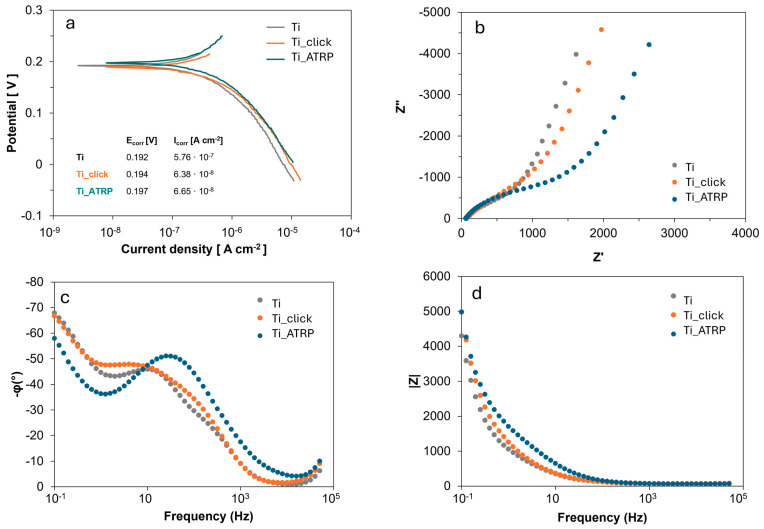
Potentiodynamic polarization curves (**a**), Nyquist (**b**) and Bode plots (**c**,**d**) from electrochemical impedance spectroscopy (EIS) analyses of coated samples compared to untreated titanium (reference).

Titanium is renowned for its excellent corrosion resistance, primarily due to the spontaneous formation of a passive film of titanium oxide (TiO_2_). Surface functionalization with polymers can significantly alter both corrosion resistance and interaction with the environment. Potentiodynamic polarization tests were performed to evaluate the corrosion behavior of untreated titanium and titanium surfaces functionalized with PDMAEMA via graft-to and graft-from approaches. The polarization curves obtained for the samples are shown in Figure 7. The electrochemical behavior was analyzed by estimating the corrosion current density (Icorr) and corrosion potential (Ecorr). The corrosion potentials measured for the three samples were very similar, indicating comparable thermodynamic tendencies toward corrosion initiation.

This similarity suggests that the presence of PDMAEMA layers does not significantly alter the corrosion potential of titanium under the tested conditions. However, a marked difference was observed in the corrosion current densities as reported in Figure 7. Icorr is an important parameter for evaluating the corrosion reaction kinetics, as it directly indicates the corrosion rate [27]. The reduction of Icorr by roughly one order of magnitude upon polymer functionalization demonstrates a slowdown of the corrosion process [28]. Hence, while the corrosion potential suggests a comparable susceptibility, the polymer coatings produce a substantial improvement in corrosion resistance.

This enhancement is attributed to the barrier effect of the PDMAEMA coatings, which impede the diffusion of corrosive species to the titanium surface and reduce the kinetics of anodic dissolution [29]. Alternative polymeric materials, including polydopamine, peptides, and chitosan, have shown similar effectiveness in enhancing corrosion resistance by forming protective layers that interact with the underlying oxide [27,30]. The comparable Icorr values between the two grafting methods indicate that both functionalization strategies effectively form protective polymer layers, despite differences in polymer architecture and grafting density. This outcome may be attributed to the short polymer chains obtained in both cases, which might limit the influence of structural differences on the corrosion behavior, rendering them less discernible under the present experimental conditions.

Electrochemical impedance spectroscopy (EIS) was performed to further investigate the electrochemical behavior of the samples. All samples exhibit a similar trend in the Nyquist plots, with overlapping depressed semicircles in the low-frequency region, suggesting that the dominant electrochemical processes are comparable across the different surfaces. The Ti_ATRP surface exhibits additional interfacial complexity, as evident in the multi-step impedance response and the shifted Warburg region. The intermediate region may correspond to the formation of an additional double layer or a distinct dielectric region at the interface between the dense polymer brush and the electrolyte. The shift of the Warburg tail to lower frequencies implies slower ion penetration through the polymer layer. Accordingly, the phase angle in the Bode plot for Ti_ATRP exhibits a broad and well-defined plateau near −80°, accompanied by a shift toward higher frequencies, indicating a more capacitive behavior and a change in the dielectric or thickness properties of the layer [31]. These observations align with the more compact and better conformationally ordered brushes typically achieved in surface-initiated polymerizations, which could impede ion mobility compared to the graft-to film. Reasonably, EIS, due to its higher sensitivity to surface and interfacial processes, can reveal subtle differences attributable to the different grafting densities.

Overall, PDMAEMA coatings, regardless of grafting method, do not introduce substantial additional resistance at the interface under these conditions. This could be attributed to the relatively thin polymer layers used in this study, which do not modify the bulk electrochemical properties of the titanium substrate. The functionalization strategies employed here preserve the essential electrochemical accessibility of the titanium surface. This is advantageous for applications where maintaining efficient charge transfer is important, such as in electrodes for sensors or biomedical devices, while still benefiting from the enhanced corrosion resistance provided by the polymer layer.

## 4. Conclusions

In this work, two strategies for functionalizing titanium surfaces with PDMAEMA polymers were developed and systematically compared: the graft-to method via click chemistry and the graft-from method based on surface-initiated ATRP. Both approaches successfully yielded uniform and stable polymer coatings, as confirmed using comprehensive chemical and morphological characterization. The surface pretreatment step emerged as a critical factor in determining grafting efficiency, directly impacting the density, distribution, and conformation of the polymer chains. Morphological analyses (SEM and AFM) revealed consistent differences in surface topography between the two grafting strategies, reflecting both the influence of the initial surface activation and the resulting polymer brush architecture. From an electrochemical standpoint, both functionalized surfaces exhibited enhanced corrosion resistance relative to bare titanium, with a notable reduction of approximately one order of magnitude in corrosion current, while maintaining comparable corrosion potentials.

Although further studies involving longer polymer chains and diverse operational conditions are required to fully optimize performance, the present findings provide valuable insights into the design of functional polymer–metal interfaces. Given the versatility of PDMAEMA brushes and their responsiveness to environmental stimuli, such functionalized surfaces hold promises for integration into micro- and nanoscale devices where controlled interfacial properties, such as corrosion resistance, wettability, or ion exchange, are critical. Potential applications may include electrochemical microsystems, biomedical interfaces, and components in responsive or adaptive microtechnologies, where finely tuned surface properties play a central role in device performance and durability.

## Data Availability

The original contributions presented in this study are included in the article. Further inquiries can be directed to the corresponding author.
